# The Complex Surgical Management of Vertebral Fracture in a Patient With Undiagnosed Ankylosing Spondylitis: A Case Report

**DOI:** 10.7759/cureus.109797

**Published:** 2026-05-28

**Authors:** Ihssan El Hak Tazi, Ismail Tazi, Inaam El Hassan Bazzout, Nabil Bouzoubaa, Tarek Mesbahi

**Affiliations:** 1 Department of Emergency Medicine, Moroccan Red Crescent Clinic, Tetouan, MAR; 2 Department of Neurosurgery, Mohammed First University, Oujda, MAR; 3 Department of Neurosurgery, Brain and Spine Care, Casablanca, MAR; 4 Department of Neurosurgery, International Clinic of Tangier, CIM Group, Tangier, MAR

**Keywords:** ankylosing spondylitis, complex spine surgery, spinal lesions, spinal trauma, vertebral fractures

## Abstract

Ankylosing spondylitis (AS) is a chronic inflammatory disorder that primarily affects the spine, resulting in progressive stiffness. Structural spinal changes increase susceptibility to fractures, even from minor trauma. These fractures are often unstable and can lead to neurological complications, posing significant challenges in surgical management. We present the case of a 60-year-old man who developed quadriplegia following a fall. Imaging revealed a displaced T3 vertebral fracture with spinal cord compression and findings consistent with AS. The patient underwent surgical stabilization with pedicle screws and interbody cage placement. Despite adequate spinal cord decompression, no postoperative neurological improvement was observed. This case highlights the increased fracture risk in AS and the complexities associated with its management.

## Introduction

Ankylosing spondylitis (AS) is an uncommon chronic inflammatory disease that primarily affects the spine and adjacent tissue structures [1]. It is characterized by persistent inflammation of the axial skeleton [2]. In its late stages, AS is commonly associated with ligament calcification and fusion of the vertebral bodies, resulting in the characteristic "bamboo spine" appearance [3]. These structural changes lead to spinal stiffness, reducing the spine's ability to bear normal loads and significantly increasing its susceptibility to fractures, even from low-energy trauma [2]. This heightened vulnerability raises the risk of vertebral displacement and severe neurological injuries [4].

Surgical management of thoracic spine fractures in AS patients facilitates stable fracture fusion, improves neurological function, and alleviates pain [5]. Despite its rarity as a rheumatological disease, AS is underreported in the literature, particularly regarding the patterns of spinal fractures and the complications associated with their surgical management [2]. This case underscores both the increased fracture susceptibility in AS patients and the significant surgical challenges encountered when managing severe spinal injuries.

## Case presentation

We present the case of a 60-year-old man who arrived at the emergency department in January 2025 with quadriplegia after sustaining trauma following a fall from standing height. He was transported on a rigid spine board with a cervical collar, in accordance with spinal precautions. The patient reported a fall from his own height with direct impact on his back, followed by the acute onset of upper back pain and inability to move his limbs.

On admission, the patient was hemodynamically stable with a blood pressure of 120/80 mmHg, a heart rate of 80 beats per minute, a respiratory rate of 16 breaths per minute, and an oxygen saturation of 98% on room air. He displayed no signs of cerebral trauma and was fully conscious, with correct eye and verbal responses. Neurological examination revealed complete quadriplegia with muscle strength graded as 0/5 in both upper and lower limbs using the Medical Research Council (MRC) Muscle Power Scale. Reflexes were absent, and sensory loss was noted below the T3 dermatome level.

A whole-body computed tomography (CT) scan performed in the emergency department revealed a distraction fracture of T3 with compression of the vertebral body, reducing the vertebral height by more than two-thirds. CT also showed posterior wall retropulsion as an osseous-density fragment, causing marked narrowing of the spinal canal and compression of the spinal cord. In addition, CT demonstrated high-density calcified bony bridges between the thoracic vertebrae, associated with high-density calcification of the anterior and posterior vertebral ligaments and diffuse demineralization of the vertebral bone matrix, giving the typical "bamboo spine" appearance suggestive of underlying AS. Magnetic resonance imaging (MRI) confirmed the severity of the compression and showed intramedullary hyperintensity on T2-weighted sequences above and below the lesion, consistent with spinal cord injury/edema (Figure 1).

**Figure 1:**
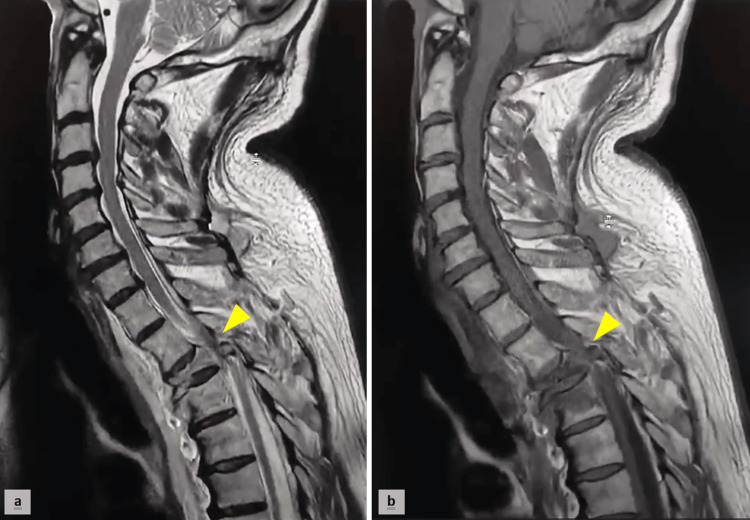
Sagittal magnetic resonance images of the spine demonstrating a D3 vertebral body fracture with spinal cord transection: (a) T2-weighted image and (b) T1-weighted image

Laboratory investigations showed normal hemoglobin level at 13.5 g/dL (normal range: 13-16 g/dL), normal white blood cell count of 7,000/µL (normal range: 4,000-10,000/µL), normal platelet count of 250,000/µL (normal range: 150,000-450,000/µL), and elevated C-reactive protein (CRP) at 75 mg/L (normal range: <5 mg/L) (Table 1).

**Table 1:** Laboratory results

Parameter	Value	Normal range
C-reactive protein	75 mg/L	<5 mg/L
Hemoglobin	13.5 g/dL	13-16 g/dL
White blood cells	7,000/µL	4,000-10,000/µL
Platelets	250,000/µL	150,000-450,000/µL
Creatinine	0.9 mg/dL	0.6-1.2 mg/dL

The patient underwent surgical management in the prone position. A posterior-only approach was performed. Pedicle screw fixation was carried out at C7, T1, T2, T4, T5, and T6, followed by transpedicular corpectomy of T3 after drilling both pedicles. The objective was to obtain spinal cord decompression and 360-degree stabilization through a single posterior approach. Fluoroscopic guidance confirmed appropriate alignment of the superior and inferior endplates. An interbody cage was then inserted, with fluoroscopic imaging confirming satisfactory positioning. The surgical procedure was complex because of the ankylosed spine, paravertebral calcification, vertebral bone hyperplasia, narrow intervertebral spaces, and poor vertebral bone quality. These factors made exposure, pedicle identification, drilling, decompression, and cage placement technically demanding. The posterior approach allowed direct decompression while avoiding an additional anterior approach.

Postoperatively, the patient received intravenous analgesics, thromboprophylaxis, and strict bed rest. On postoperative day 1, the neurological assessment revealed no improvement, with persistent muscle strength of 0/5 in all limbs. A follow-up MRI confirmed spinal cord decompression, and 3D reconstruction demonstrated satisfactory pedicle screw positioning (Figure 2).

**Figure 2:**
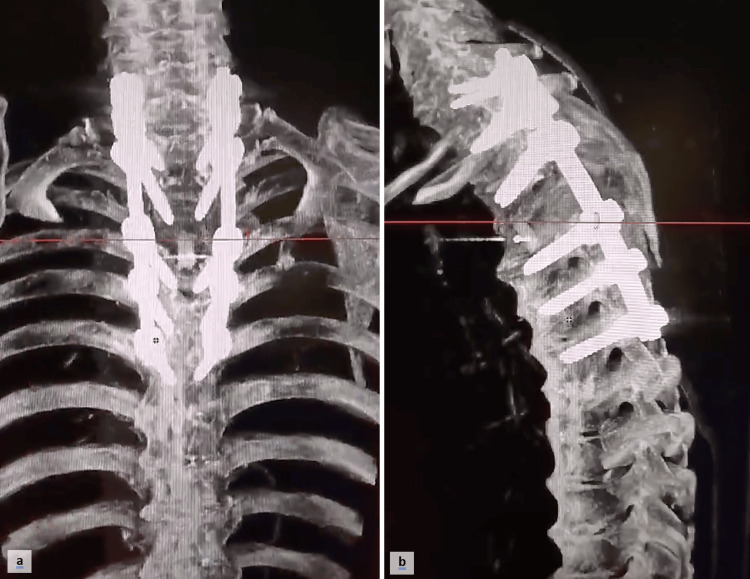
Postoperative 3D reconstructed magnetic resonance imaging showing satisfactory outcomes with pedicle screws in place: (a) en face view and (b) lateral view

Given the high clinical and radiological suspicion of AS, HLA-B27 testing was performed and returned positive, further supporting the diagnosis. The diagnosis was retained based on chronic inflammatory back pain, typical radiological findings with bamboo spine appearance, and HLA-B27 positivity. The patient was transferred to a physical rehabilitation unit for specialized rehabilitation exercises and regular follow-up, with long-term rheumatologic management planned.

During follow-up, one month after admission, the patient developed respiratory distress requiring brief hospitalization in the intensive care unit, with no evident cause identified. He fully recovered from this episode. Unfortunately, one month later, he experienced his first cardiac arrest from which he recovered, followed by a second irreversible cardiac arrest. Radiological investigations revealed massive pulmonary embolism with bilateral pleural effusion, which was identified as the cause of the fatal outcome.

## Discussion

AS is an uncommon, chronic, and debilitating rheumatic disorder primarily affecting the axial skeleton, characterized by chronic inflammation of the spine [6]. Its estimated annual incidence ranges from 0.5 to 14 cases per 100,000 people [2]. The etiology of AS remains poorly understood, with multiple factors implicated, including genetic, autoimmune, and environmental components. It predominantly affects young adults, with a male predominance [5]. The disease is marked by inflammation of the vertebral joints and ligaments, leading to vertebral fusion and stiffening of the intervertebral spaces, which reduces spinal flexibility [6]. Furthermore, this inflammation contributes to bone resorption, increasing bone fragility and predisposing patients to osteoporosis [7]. Most patients with AS show positivity for the HLA-B27 antigen [8].

Clinically, AS manifests as back pain, stiffness, kyphosis, reduced chest expansion, peripheral arthritis, and enthesopathy. These deformities exacerbate balance issues, heightening the risk of falls [3]. Extra-articular manifestations include cardiovascular, respiratory, renal, and ocular complications, which are also common [2]. The risk of vertebral fractures increases with disease severity and progression due to diminished spinal stability and stiffness [9]. These fractures are often unstable, posing a significant risk of neurological injury, including quadriparesis, quadriplegia, paraparesis, or paraplegia. Additionally, the instability can lead to iatrogenic spinal cord injuries during unprotected transfers, underscoring the importance of immobilizing these patients in their natural posture [5].

The fracture level varies based on the trauma mechanism and impact nature. Kandregula et al. reported that thoracic fractures were most common (58%), followed by cervical fractures (38%), with multiple fractures being less frequent. Spinal cord injury occurred in 15.8% of patients, primarily associated with cervical fractures [2]. Backhaus et al. similarly found predominance of thoracic fractures (42.6%) [10]. However, other large case series, including Teunissen et al. (60.8%), Lukasiewicz et al. (53%), and Robinson et al. (53.9%), reported cervical fractures as the most prevalent [11-13].

Thorough spinal imaging is essential in AS patients following even minor trauma to prevent undetected fractures, especially when sudden spinal pain or postural changes occur [14]. CT scans are recommended for accurate fracture assessment, while MRI is reserved for suspected soft tissue lesions, spinal cord injuries, or epidural hematomas. Syndesmophyte formation results in the characteristic bamboo spine appearance, as seen in our patient. Comprehensive evaluation of the entire spine is advised in suspected fractures [5]. In our case, the post-trauma onset of quadriplegia strongly suggested spinal cord injury, prompting MRI, which revealed a T3 fracture with spinal cord compression and confirmed the diagnosis of AS.

Non-surgical immobilization is often ineffective; surgical management is recommended due to frequent neurological impairments, necessitating posterior stabilization with long constructs spanning at least three vertebral levels above and below the fracture for adequate stress distribution [5]. In patients with sagittal kyphosis, the prone position during surgery can exacerbate the distraction-extension mechanism, worsening deformities. Surgical fixation is further complicated by the increased bone fragility and altered bone and ligament structures in AS, requiring meticulous planning and execution [5]. The fracture location typically dictates the surgical approach, with posterior approaches preferred for better alignment, stability, and decompression of neural structures. The anterior fixation approach is associated with higher failure rates [15]. Patients with AS and spinal fractures face higher postoperative complication rates than the general population. Common complications include ventilator dependency, renal injury, urinary tract infections, and sepsis, especially in cervical fractures [2]. In our patient, spinal injury necessitated fusion surgery to decompress the spinal cord, prevent further neurological damage, and ensure long-term spinal stability.

Despite treatment, patients with AS who sustain spinal fractures face considerable morbidity and a mortality rate that can reach up to 11% [2].

## Conclusions

This case aims to emphasize the increased vulnerability of patients with AS to spinal fractures and the distinct challenges associated with their surgical management. It underscores the critical importance of early recognition and appropriate intervention for spinal fractures in the context of bone fragility and spinal deformities inherent in these patients. Despite treatment, AS-associated spinal fractures result in considerable morbidity and mortality rates, emphasizing the need for meticulous perioperative care, long-term follow-up, and tailored rehabilitation strategies to optimize outcomes.
